# Dynamics of naturally acquired antibody against *Haemophilus influenzae* type a capsular polysaccharide in a Canadian Aboriginal population

**DOI:** 10.1016/j.pmedr.2016.01.004

**Published:** 2016-01-26

**Authors:** Angjelina Konini, Eli Nix, Marina Ulanova, Seyed M. Moghadas

**Affiliations:** a Agent-Based Modelling Laboratory, York University, 4700 Keele St., Toronto, Ontario M3J 1P3, Canada; b Northern Ontario School of Medicine, Lakehead University, Thunder Bay, Ontario, Canada

**Keywords:** *Haemophilus influenzae* a (Hia), Invasive disease, Antigenic challenge, Serum assay, Capsular polysaccharide, Mathematical model, Descriptive statistics, Simulation

## Abstract

Severe infections caused by *Haemophilus influenzae* type a (Hia) have reached alarming rates in some Canadian Aboriginal communities. We sought to estimate the frequency of exposure to this pathogen and timelines for boosting protective antibodies.

We developed a model of secondary antigenic challenge (natural exposure), and used data for anti-Hia antibodies in serum samples of healthy and immunocompromised adults in a population of Northwestern Ontario, Canada. We parameterized the model with available estimates from previous studies for the decay rate of antibody and its protective levels against both Hia carriage and invasive disease. Simulations were initialized using antibody concentrations from data. We investigated both the duration of immunity without secondary antigenic challenge and the average time between subsequent exposures to Hia.

When there was no new natural exposure, serum antibody concentrations in healthy Aboriginal individuals decreased below the level (1 μg/ml) assumed for protection against invasive Hia disease 3 years after primary exposure. This period was shorter (about 2 years) for Aboriginal individuals suffering from chronic renal failure. We estimated that a new antigenic challenge occurs once in 5 and 2 years for healthy and immunocompromised Aboriginal individuals, respectively. More frequent natural exposure was required to maintain protective antibody levels for non-Aboriginal individuals compared to Aboriginal individuals.

The findings suggest that frequent boosting of natural immunity is required to maintain the anti-Hia antibody levels protecting against invasive Hia disease, particularly in individuals with underlying medical conditions. This information has important implications for immunization when an anti-Hia vaccine becomes available.

## Highlights

• Frequent natural exposure to Hia is required to maintain protective immunity.
• Epigenetic factors may affect formation of natural immunity in Aboriginal people.
• Further data pertaining to immunological correlates of Hia are needed.
• Modeling can inform timelines of boosting immunity and vaccination policies.

## Background

*Haemophilus influenzae* type a (Hia) is an important bacterial pathogen, which can cause severe invasive disease (Ulanova and Tsang, 2014). The invasive disease manifestation occurs mainly in young children (younger than 2 years of age), the elderly, and immunocompromised individuals (Ulanova, 2013). Hia is one of the 6 known encapsulated strains of *H. influenzae* classified based on their distinct capsular antigens (Ryan and Ray, 2004). *H. influenzae* serotype b (Hib) was the major cause of bacterial meningitis in young children worldwide before the introduction of an effective conjugate Hib vaccine in the late 1980s (Adams et al., 1993). Routine vaccination against Hib dramatically decreased the incidence of invasive Hib disease and carriage of the pathogen in the countries where vaccination programs were implemented (Ulanova and Tsang, 2009). Since protection conferred by the Hib vaccine is specific to the type b polysaccharide capsule, widespread vaccination against Hib may have unmasked the disease caused by other serotypes (Lipsitch, 1999).

The preceding decade has witnessed the emergence of Hia as the dominant encapsulated strain of *H. influenzae* in several specific geographic locations and populations, including Aboriginal populations in North America (Rotondo et al., 2013, Bruce et al., 2013). Clinical and epidemiological studies of Hia indicate that Aboriginal children (younger than 5 years of age) and adults with predisposing medical conditions are most affected by invasive Hia disease (Ulanova, 2013). While currently there is no vaccine to prevent Hia infection, understanding of the immunological and epidemiological characteristics of this pathogen is imperative to develop preventive measures with long-lasting effects. Similar to Hib, since vaccination with a conjugate vaccine specific to Hia could reduce the circulation of Hia bacteria, and therefore the incidence of Hia carriage in the population, determining timelines for boosting of protective immunity will be essential for maintaining a high level of herd immunity (Konini and Moghadas, 2015). In case of Hib, the frequency of new exposure required to maintain protective serum antibody concentrations ≥ 1.0 μg/ml was estimated at a minimum of 1 in 4 years prior to the introduction of conjugated Hib vaccine (Leino et al., 2000). Currently, there are no estimates on timelines for Hia recurrent natural exposure and boosting of antibody concentration.

In this study, we developed a model of secondary antigenic response using data for anti-Hia antibody concentrations in the serum samples of the participants in a population of Northwestern Ontario, Canada. Our aim was to estimate the timelines for boosting antibody concentrations following priming for different populations' characteristics. We considered the age, sex, ethnicity (Aboriginal and non-Aboriginal), and health status (including those presenting chronic renal failure) of individuals in stratifying collected data. Since the rates of antibody decay against Hia are still unknown, we parameterized the model using available estimates from the published studies for Hib. We based this assumption on the similarities between Hia and Hib capsular polysaccharide antigens (Branefors-Helander, 1977, Crisel et al., 1975).

## Materials and methods

Our methodology included sample collection and laboratory assays, data analysis, model development, simulation experiments and sensitivity analysis of the model outcomes. Details of laboratory assays are provided in the Appendix. Data collection and analysis were approved by the Thunder Bay Regional Health Sciences and Lakehead University research ethics boards.

### Study population

This study is based on the analysis of Hia seroprevalence data in a population of Northwestern Ontario, Canada, which is characterized by a presence of a significant proportion of indigenous people, i.e., 19.6% of the total population in this area (Statistics Canada, 2006). In comparison, according to the 2006 Canadian Census, indigenous people comprise 3.8% of the Canadian population (Anon., 2006). Healthy adults aged 19–80 years who self-identified as either Aboriginal or non-Aboriginal were recruited from the Thunder Bay area. Individuals with chronic renal failure (CRF) aged 24–91 years were recruited from the Renal Services, Thunder Bay Regional Health Sciences Centre. The characteristics of the study groups are presented in a recently published study (Nix et al., 2015). Table 1 summarizes the demographic and health status of the participating subjects.

In designing and conducting this research, we have fully adhered to the core principles of ownership, control, access, and possession as defined by the National Aboriginal Health Organization and the Tri-Council Policy Statement on Ethical Conduct for Research Involving Humans, specifically those outlined in Chapter 9 of the ‘Research Involving the First Nations, Inuit and Métis Peoples of Canada’ (Anon., 2015, Schnarch, 2004). In particular, prior to the beginning of the study we engaged in an extensive consultation with a variety of stakeholders and received letters of endorsement from several regional Aboriginal organizations including the Nishnawbe Aski Nation (political territorial organization representing 49 First Nation communities in Northern Ontario), the Métis Nation of Ontario, the Red Rock Indian Band (Lake Helen First Nation), the Bingwi Neyaashi Anishinaabek (Sand Point First Nation), and the Fort William First Nation. During the data collection phase, we have regularly updated our Aboriginal partners through information sessions and progress reports as results are analyzed.

### Antibody concentration

**Fig. 1:**
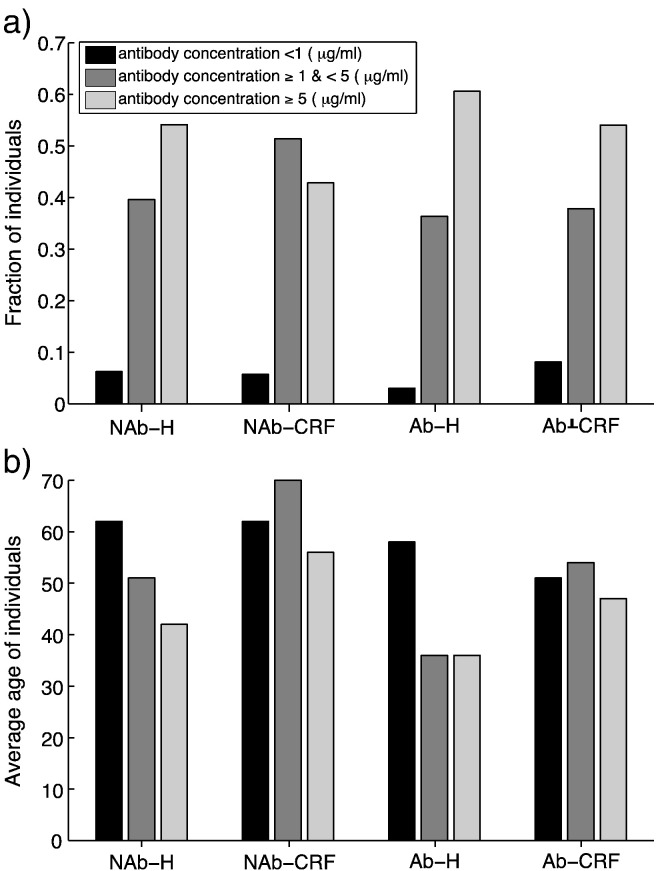
(a) The fraction of individuals at low risk of infection (AC ≥ 5 μg/ml; light grey), low risk of invasive disease (1 μg/ml ≤ AC < 5 μg/ml; dark grey); and high risk of invasive disease (AC < 1 μg/ml; black). (b) The corresponding average ages of individuals identified as healthy Non-Aboriginal (NAb-H); Non-Aboriginal with chronic renal failure (NAb-CRF); healthy Aboriginals (Ab-H); and Aboriginals with chronic renal failure (Ab-CRF). (AC: total IgG and IgM antibody concentration).

On the basis of previous findings on immunological correlates of protection against Hib disease (Leino et al., 2000, Eskola et al., 1985, Käyhty et al., 1983), we hypothesized that anti-Hia polysaccharide antibody concentrations of 1 μg/ml or above provide long-term protection against invasive Hia disease and concentrations of 5 μg/ml or above prevent colonization of the upper airways. We stratified the collected data for the level of antibody concentrations and the corresponding average ages of individuals in Fig. 1. In all categories of healthy and CRF participants, the level of antibody concentration < 1 μg/ml corresponds to the lowest fraction of individuals, with an average age older than 50 years.

### The model

We first used logistic regression to investigate the effect of age and sex on the concentration of anti-Hia capsular polysaccharide antibody in the serum samples of the study participants. The statistical significance of adding the interaction term for age and sex was assessed by using a likelihood ratio test. We performed this analysis for both Aboriginal and non-Aboriginal participants. All tests were at two-sided significance level of 0.05. The results of this analysis indicated that variables of sex and age were not significant on the level of antibody concentration, suggesting that the antibody-boosting model could be developed independent of these variables.

#### Antibody boosting

We assumed that in the absence of any boosting, the initial antibody level decays in the exponential form *c*(*t*) = *c*_0_*e*^−* k*(*t* − *t*_0_)^*a*^^, where *c*(*t*) is the antibody concentration at time *t*; *c*_0_is the initial concentration, *k* is the decay rate (per time); and *a* is the parameter regulating the antibody decline in the range 0–1 (Auranen et al., 1999).

When stimulating challenge occurs after time *t*_0_, the antibody concentration is increased in the process of boosting immune responses. The level of boosting, however, depends on *c*(*t*) at the time of natural exposure. Consistent with previous work (Ulanova and Tsang, 2014, Chokephaibulkit et al., 2004, Concepcion and Frasch, 1998, Hawdon et al., 2012), we considered two threshold levels of antibody concentrations:

i) Subclinical threshold (*L*_1_): concentration above this level prevents the development of subclinical disease (carriage) following exposure, and a minimal stimulation of the immune system may occur.
ii) Invasive threshold (*L*_2_): concentration above this level, but below *L*_1_ is not adequate for infection protection. However, this level of antibody concentration will mitigate infection if occurs, and prevent invasive form of disease. Exposure to infection during the period in which *L*_2_ < *c*(*t*) < *L*_1_ leads to a moderate boosting of the immune response and a measurable increase in the level of antibody concentration.

The maximum level of boosting occurs when *c*(*t*) < *L*_2_, especially when the individual is naïve. Due to the possibility of boosting immunity following priming, we enhanced the exponential decay model to include the increase in the level of antibody concentration. We considered *c*^′^(*t*) = − *kc*(*t*) + *b*(*t*), where *b*(*t*) is the boosting rate at the time of natural exposure. This rate depends on several key parameters, and we used the Heaviside step function to describe the increase in *c*(*t*) (Fowler, 1981). We defined

$$\theta \left(t\right)=\frac{\beta \left[H\left(L_{1}-c\left(t\right)\right)+H\left(L_{2}-c\left(t\right)\right)\right]}{1+H\left(L_{1}-c\left(t\right)\right)},$$

where *β* is the maximum boosting rate in the absence of pre-existing immunity. Denoting the average time for a secondary natural exposure following priming by *τ*, we defined *b*(*t*) = *θ*(*t*)*δ*(*t* − *nτ*), for *n* = 1 , 2 , ⋯, where Kronecker *δ* is defined by:

$$\delta \left(t-\mathrm{n\tau}\right)=\left\{\begin{array}{cc} 1 & \text{if}\;t=\mathrm{n\tau}\\ 0 & \text{if}\;t\neq \mathrm{n\tau}\; \end{array}\right)$$

This functional form of boosting allows for the increase in the antibody concentration based on the amount of *c*(*t*) at the time of natural exposure. We estimated the rate of boosting *β* = *c*_*m*_ as a result of new natural exposure following priming, where *c*_*m*_ is the geometric mean concentration (GMC) of the measurements for antibody concentration. For antibody concentrations below *L*_2_, the magnitude of simulated boosting response was full at the time of exposure (that is similar to the case where there is no preexisting immunity). This magnitude reduced by 50% (as defined in *θ*(*t*)) for antibody concentrations between *L*_2_ and *L*_1_ at the time of exposure.

### Parameterization and simulations

We parameterized the model using available estimates from the published studies for Hib. In the absence of vaccination, the duration and the decline rate of antibody concentration depend on the incidence of infection in the population (Leino et al., 2000). We assumed a decay rate in the range of 0.08–0.16 per year (converted to a rate per month) (Leino et al., 2000), and used antibody concentration levels of *L*_1_ = 5 and *L*_2_ = 1 (μg/ml) for preventing subclinical infection and invasive disease, respectively. Previous work pertinent to Hib indicates that individuals with antibody concentrations above 1 μg/ml were protected against invasive disease despite the occurrence of infection (Leino et al., 2000, Eskola et al., 1985). The Latin Hypercube Sampling (LHS) technique was used to evaluate the effect of simultaneous variation of model parameters on the outcomes.

### Parameter variation and sampling

To determine the timelines for decline of antibody concentration, we used the Latin Hypercube Sampling (LHS) technique (McKay et al., 1979) and ran simulations for a 10-year period following priming, when *k* and *a* are given by LHS. To allow for the simultaneous variations of these parameters, samples of size *n* = 100 were generated in which each parameter was treated as a random variable and assigned a probability function. These parameters were uniformly distributed and sampled within their respective ranges. Each parameter set was simulated using the total IgG and IgM antibody concentrations measured for each individual in the collected samples as the initial condition at time *t*_0_. For each scenario, corresponding to the average number of years for a new natural exposure, we ran 1000 independent simulations, and determined the geometric mean concentration (GMC) and 95% confidence intervals of the antibody concentration within 10 years.

## Results

### Duration of immunity without secondary antigenic response

**Fig. 2:**
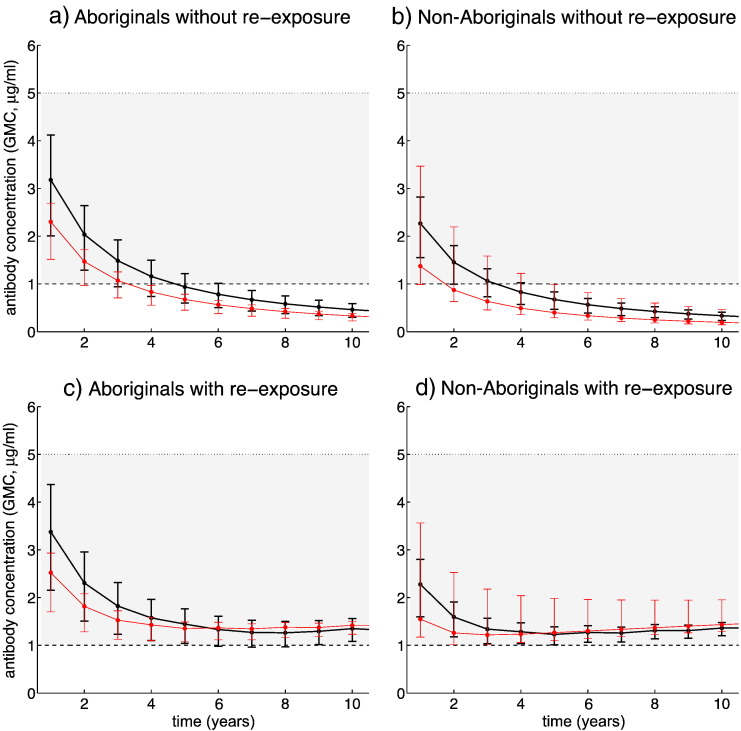
The median GMC antibody levels with their predictive 95% confidence intervals over a 10-year time period following priming, without (a and b) and with a new natural exposure (c and d). The risk of carriage is shown in grey area. Black and red curves correspond to healthy and CRF subjects, respectively. For Aboriginals (panel c), new natural exposure occurred once in 5 years for healthy individuals (black curve) and once in 2 years for individuals with CRF condition (red curve). For non-Aboriginals (panel d), new exposure occurred once in 2 years for healthy individuals (black curve) and once every year for individuals with CRF condition (red curve).

For scenarios in which there is no new natural exposure, we observed that the level of antibody concentration in healthy Aboriginal individuals would be expected to fall below the level (1 μg/ml) assumed for protection against invasive disease 3 years after priming (Fig. 2a, black curve). The period of protection against invasive disease is shorter (2 years) for immunocompromised Aboriginal individuals suffering from CRF (Fig. 2a, red curve). The corresponding protection periods against invasive disease for non-Aboriginals are 2 years (for healthy individuals) and 1 year (for individuals with CRF condition), as shown in Fig. 2b.

### Average time between exposures

We simulated the model to estimate the average time within which a new natural exposure will need to occur in order to maintain the population level of antibody concentration above 1 μg/ml. For Aboriginal individuals, we estimated this average time to be 5 and 2 years for healthy and CRF subjects, respectively (Fig. 2c and d). For healthy non-Aboriginals, a new natural exposure was estimated to occur once in 2 years. We estimated a higher frequency (once every year) of new natural exposure to prevent the risk of invasive disease in non-Aboriginals with CRF condition.

## Discussion

In this study, we made a systematic attempt to develop a model that can be used to estimate timelines for boosting protective antibody concentrations following priming. Our results, based on parameter estimates for Hib, indicate that Hia colonization may be occurring more frequently in some populations, such as North American indigenous people, compared to previous estimates for Hib (Leino et al., 2000). Indeed, recent studies indicate that Aboriginal adults have significantly higher serum bactericidal activity against Hia compared to non-Aboriginal individuals living in the same geographic area suggesting an increased exposure to the pathogen in the former population (Nix et al., 2015). Although current rates of Hia colonization in the North American population and immunological correlates of protection against this pathogen are unknown, our data suggest that adult serum antibody concentrations are above the threshold required for the prevention of Hia invasive disease in the Aboriginal populations of Northwestern Ontario (Canada). Our model indicates that frequent natural exposure is required to maintain the anti-Hia capsular polysaccharide antibody levels against invasive Hia disease, particularly in individuals with chronic renal failure who are immunocompromised as a result of profound metabolic consequences of uremia, underlying medical conditions (e.g., type 2 diabetes mellitus), and hemodialysis procedure (Betjes, 2013).

Severe infections caused by Hia have reached alarming rates in terms of morbidity in Canadian Aboriginal communities (Ulanova and Tsang, 2014). The case-fatality rate of invasive Hia disease among pediatric cases reported by Canadian IMPACT centers in 1996–2001 reached 16% (McConnell et al., 2007). From 2000 to 2010, 56% of identified cases of invasive *H. influenzae* disease with serotype information were caused by Hia in Canadian northern populations, with average invasive Hia disease of 4.6 cases per 100,000 population per year over these 11 years (Rotondo et al., 2013). The reasons behind the vulnerability of Aboriginal people to invasive Hia disease are unknown, but could be related to biological and environmental factors, both requiring a systematic study. In the pre-Hib vaccine era, the indigenous populations of North America and Australia experienced the highest incidence rates of invasive Hib disease worldwide (Ward et al., 1981, Losonsky et al., 1984, Hansman et al., 1986, Hanna, 1990). However, the experience with Hib vaccines over the past 20 years has demonstrated that even highly vulnerable populations can be successfully protected using immunization with protein–polysaccharide conjugated vaccine (Santosham et al., 1992, Singleton et al., 2006). Therefore, the immunization of vulnerable populations against Hia may be a solution to reduce incidence, and possibly control Hia disease in affected communities.

Immunization can induce indirect protection through herd immunity, which reduces pathogen transmission in the population. This can have a profound impact on immunity at the individual level, as protective antibody titers decline over time without boosting, which will in turn affect the levels of herd immunity. Our findings in this study highlight the importance of vaccination and timely boosting of the individual's immunity within the expected duration of vaccine-induced protection against Hia. Such information on the required frequency of boosting immunity is essential for the development of vaccination policies that have long-lasting effects on curtailing Hia incidence (Konini and Moghadas, 2015).

Our study has several limitations that highlight the need for further investigations and data collection in specific population settings. First and foremost is the fact that we parameterized our model with the range of antibody decay previously estimated for Hib prior to vaccination. However, to estimate the Hia-specific decay rates of antibody concentrations in the absence of vaccination, subsequent measurements of serum samples should be collected for several years. While such data can provide better estimates of timelines for boosting concentrations, we note that antigens from several organisms other than Hia can also induce cross-reactive antibodies to Hia capsular polysaccharide (Lagergard and Branefors, 1983, Fekete et al., 2006), and may therefore increase the antibody concentration. Since the relative importance of antibodies arising in response to encounters with cross-reactive bacteria is unknown, the measurements of the serum Hia antibody concentration may not be reliable in detecting carriage. We assumed the same subclinical infection and invasive disease thresholds for both Aboriginal and non-Aboriginal populations. However, given the reported incidence of Hia in the northern communities of Canada (Ulanova and Tsang, 2014, Rotondo et al., 2013, McConnell et al., 2007), it may be the case that these thresholds are higher for Aboriginal populations compared to non-Aboriginal populations. These considerations merit further investigation.

Nevertheless, it is important to recognize that self-identification as Aboriginal comes with significant admixture and that biological or genetic differences are unlikely to play a significant role in accounting for the current epidemiology of invasive Hia disease. The social determinants of health, which Aboriginal people have had to endure, are very likely to be a major factor combined with the geographical characteristics of the population. The city where the data have been collected (Thunder Bay, Ontario) is a hub for the region and many Aboriginal people living/working in the city regularly visit their home communities, which may lead to increased exposure. These factors may potentially explain the differences in antibody concentrations between the groups.

## Conclusions

The measurements of antibody concentration in the data used for this study show that a significant fraction of participants have anti-Hia antibody levels above 1 μg/ml. Our model suggests that frequent boosting of immunity through natural infection is required to maintain protective antibody levels above 1 μg/ml observed in the data. Among factors that could affect the incidence rate of Hia carriage and disease, and therefore herd immunity in the population, are socio-economic and environmental conditions, particularly in Canadian northern communities with predominantly Aboriginal populations (Tsang et al., 2014). These factors warrant further investigation for understanding the biological and epidemiological mechanisms responsible for high prevalence of Hia circulation in these population settings. Although genetic differences between Aboriginal and non-Aboriginal populations may be obscured by admixture, the role of epigenetic factors associated with social determinants of health may be more important. Understanding of these complex factors will be essential for implementing immunization and booster strategies (Konini and Moghadas, 2015) with long-lasting effects when an anti-Hia vaccine candidate becomes available.

## Conflict of interest statement

The authors declare that there are no conflicts of interests.

## Authors' contributions

AK analyzed the data, developed the simulation model, performed the experiments, and substantially contributed to writing the manuscript. EN and MU collected the data, contributed to writing the manuscript and interpretation of the results. SM conceived the study and simulation model, contributed to writing the manuscript, and provided important intellectual content. All authors have read the final version of this manuscript and approved it.

## Transparency Document

Transparency document.

## Figures and Tables

**Table 1:** Summary of the data collected based on the analysis of Hia seroprevalence in a population of Northwestern Ontario, Canada.

Age/Sex	Aboriginal				Non-Aboriginal			
Healthy		CRF		Healthy		CRF	
F	M	F	M	F	M	F	M
19–34	29	5	2	4	10	7	2	0
35–59	23	5	14	7	13	7	4	4
≥ 60	3	1	8	2	5	6	6	19
All	55	11	24	13	28	20	12	23

F: female; M: male; CRF: chronic renal failure.
