# Supplementary material for: Dynamics of naturally acquired antibody against Haemophilus influenzae type a capsular polysaccharide in a Canadian Aboriginal population
Source: Prev Med Rep. 2016 Jan 26;3:145–50. doi: 10.1016/j.pmedr.2016.01.004 (PMC4929236; doi:10.1016/j.pmedr.2016.01.004)
Supplement: Supplementary file 2 — Supplementary material [file mmc1.docx]

**Appendix: Details of Laboratory Assays**

**Dynamics of naturally acquired antibody against *Haemophilus influenzae* type a capsular polysaccharide in a Canadian Aboriginal population**

**Angjelina Konini**

*Agent-Based Modelling Laboratory, York University, Toronto, Ontario, Canada*

**Eli Nix**

**Marina Ulanova**

*Northern Ontario School of Medicine, Lakehead University, Thunder Bay, Ontario, Canada*

**Seyed M. Moghadas**

*Agent-Based Modelling Laboratory, York University, Toronto, Ontario, Canada*

**Serum assays**

Serum samples were obtained from the participants after informed consent was given and stored at -80 °C prior to use.

*Anti-Hia capsular polysaccharide IgG ELISA*

The assay was developed based on published methodology with some modifications [^[[1]](#endnote-1)^]. The Hia capsular polysaccharide was isolated according to the method of Anderson and Smith [^[[2]](#endnote-2)^] Based on a developed methodology [^[[3]](#endnote-3)^], the isolated polysaccharide was then oxidized and conjugated to human serum albumin, and the resulting conjugate was purified and characterized [^[[4]](#endnote-4)^]. Hia polysaccharide conjugated to human serum albumin was dissolved in coating buffer (0.01M PBS) and 100 µl added to each well of a 96-well ELISA plate (Cedarlane, Burlington, Canada) at a concentration of 1 µg/ml, covered with plate sealer (Fisher Scientific, Ottawa, Canada) and incubated at 37 °C for 1.5 hours. The standard and samples were serially diluted in dilution buffer (0.01M PBS, 0.3% Tween 20) 1:400 to 1:25,600 and 1:200 to 1:1,600, respectively and run in duplicate. Plates were washed five times with 250 µl wash buffer (0.01M PBS, 1.2% Tween 20), and the first addition of wash solution was left on the plate for 1 minute. Next, 100 µl of diluted serum was added to each well and left at room temperature for 90 minutes then washed as previously described. Horseradish peroxidase conjugated mouse anti-human IgG antibody (Hybridoma Reagent Laboratory, Baltimore, MD) was diluted 1:4,000 in antibody buffer (0.01M PBS, 0.05% Tween 20), 100 µl added to each well and incubated for 2 hours at room temperature. Then 100 µl of Sure Blue TMB peroxidase substrate (Mandel Scientific, Guelph, Canada) was added to each well and incubated at room temperature for 30 minutes followed by the addition of 100 µl of 1N HCl. The colorimetric substrate was detected using a microplate reader (BioTek Powerwave XS; Winooski,VT) at 450 nm with 630 nm reference. Quantification of antibody was performed using a previously described methodology [1] The concentration of anti-Hia polysaccharide IgG (4.1 µg/ml) in the standard was determined by cross-standardization to the Hib (FDA 1983) reference serum [^[[5]](#endnote-5)^]. The quantification range was 0.10-4 µg/ml. Samples above the upper limit of quantification were diluted an additional 5 times and re-assayed effectively increasing the upper limit of quantification to 20 µg/ml.

*Anti-Hia capsular polysaccharide IgM ELISA*

To quantify anti-Hia capsular polysaccharide IgM, the anti-Hia PS IgG protocol was used with the following modifications. Following coating, the plates were blocked for 2 hours at room temperature with antibody dilution buffer containing 1% fish gelatin (Sigma-Aldrich, Oakville, Canada) and washed as described. Serum IgG was depleted using IgG/RF stripper [^[[6]](#endnote-6)^]. As a standard, we selected the serum of a volunteer (NOSM 97/13) from our collection that exhibited high bactericidal activity against Hia. Following a two-fold serial dilution scheme (1:100 to 1:6,400) serum samples were incubated with coated plates for 60 minutes. As a secondary antibody, horseradish peroxidase conjugated goat anti-human IgM (SouthernBiotech, Birmingham, AL) diluted 1:5,000 in antibody dilution buffer was used and incubated in the wells for 1 hour at room temperature. The concentration of anti-Hia PS IgM (3.84 µg/ml) in the standard was determined by cross-standardization to the Hib (FDA 1983) standard [5]. The estimated range of quantification was 0.01-18 µg/ml, for statistical purposes samples below the lower limit of quantification were assigned a value one half the lower quantification limit. Samples above the upper limit of quantification were diluted an additional 5 times and re-assayed increasing the upper limit of quantification to 90 µg/ml.

**References**

1. . Schmidt DS, Bieging KT, Gomez-de-Leon P, et al. Measurement of Haemophilus influenzae type a capsular polysaccharide antibodies in cord blood sera. *Pediatr Infect Dis J* 2012; 31: 876-878. [↑](#endnote-ref-1)
2. . Anderson P, Smith DH. Isolation of the capsular polysaccharide from culture supernatant of Haemophilus influenzae type b. *Infect Immun* 1977; 15: 472-477. [↑](#endnote-ref-2)
3. . Anderson PW, Pichichero ME, Stein EC, et al. Effect of oligosaccharide chain length, exposed terminal group, and hapten loading on the antibody response of human adults and infants to vaccines consisting of Haemophilus influenzae type b capsular antigen unterminally coupled to the diphtheria protein CRM197. *J Immunol* 1989; 142: 2464-2468. [↑](#endnote-ref-3)
4. . Cox AD, St Michael F, Neelamegan D, et al. Investigating the candidacy of LPS-based glycoconjugates to prevent invasive meningococcal disease: chemical strategies to prepare glycoconjugates with good carbohydrate loading. *Glycoconj J* 2010; 27: 401-417. [↑](#endnote-ref-4)
5. . Concepcion N, Frasch CE. Evaluation of previously assigned antibody concentrations in pneumococcal polysaccharide reference serum 89SF by the method of cross-standardization. *Clin Diagn Lab Immunol*. 1998; 5: 199-204. [↑](#endnote-ref-5)
6. . Hawdon N, Nix EB, Tsang RS, Ferroni G, McCready WG, Ulanova M. Immune response to Haemophilus influenzae type b vaccination in patients with chronic renal failure. *Clin Vaccine Immunol*. 2012; 19:967-969. [↑](#endnote-ref-6)
